# Outcome analysis of retrograde nailing and less invasive stabilization system in distal femoral fractures: A retrospective analysis

**DOI:** 10.4103/0019-5413.80043

**Published:** 2011

**Authors:** Christian Hierholzer, Christian von Rüden, Tobias Pötzel, Alexander Woltmann, Volker Bühren

**Affiliations:** Department of Trauma Surgery, Trauma Center Murnau, Murnau, Germany

**Keywords:** Distal femoral fractures, osteosynthesis, minimally invasive techniques, retrograde intramedullary nailing, angular stable plating

## Abstract

**Background:**

Two major therapeutic principles can be employed for the treatment of distal femoral fractures: retrograde intramedullary (IM) nailing (RN) or less invasive stabilization on system (LISS). Both operative stabilizing systems follow the principle of biological osteosynthesis. IM nailing protects the soft-tissue envelope due to its minimally invasive approach and closed reduction techniques better than distal femoral locked plating. The purpose of this study was to evaluate and compare outcome of distal femur fracture stabilization using RN or LISS techniques.

**Materials and Methods:**

In a retrospective study from 2003 to 2008, we analyzed 115 patients with distal femur fracture who had been treated by retrograde IM nailing (59 patients) or LISS plating (56 patients). In the two cohort groups, mean age was 54 years (17–89 years). Mechanism of injury was high energy impact in 57% (53% RN, 67% LISS) and low-energy injury in 43% (47% RN, 33% LISS), respectively. Fractures were classified according to AO classification: there were 52 type A fractures (RN 31, LISS 21) and 63 type C fractures (RN 28, LISS 35); 32% (RN) and 56% (LISS) were open and 68% (RN) and 44% (LISS) were closed fractures, respectively. Functional and radiological outcome was assessed.

**Results:**

Clinical and radiographic evaluation demonstrated osseous healing within 6 months following RN and following LISS plating in over 90% of patients. However, no statistically significant differences were found for the parameters time to osseous healing, rate of nonunion, and postoperative complications. The following complications were treated: hematoma formation (one patient RN and three patients LISS), superficial infection (one patient RN and three patients LISS), deep infection (2 patients LISS). Additional secondary bone grafting for successful healing 3 months after the primary operation was required in four patients in the RN (7% of patients) and six in the LISS group (10% of patients). Accumulative result of functional outcome using the Knee and Osteoarthritis Outcome (KOOS) score demonstrated in type A fractures a score of 263 (RN) and 260 (LISS), and in type C fractures 257 (RN) and 218 (LISS). Differences between groups for type A were statistically insignificant, statistical analysis for type C fractures between the two groups are not possible, since in type C2 and C3 fractures only LISS plating was performed.

**Conclusion:**

Both retrograde IM nailing and angular stable plating are adequate treatment options for distal femur fractures. Locked plating can be used for all distal femur fractures including complex type C fractures, periprosthetic fractures, as well as osteoporotic fractures. IM nailing provides favorable stability and can be successfully implanted in bilateral or multisegmental fractures of the lower extremity as well as in extra-articular fractures. However, both systems require precise preoperative planning and advanced surgical experience to reduce the risk of revision surgery. Clinical outcome largely depends on surgical technique rather than on the choice of implant.

## INTRODUCTION

The incidence of distal femur fractures is around 37/100,000 patients per year.^1^ Typically, two distinct mechanisms of injury cause distal femur fractures. In the older population with osteoporotic bone and vulnerable soft-tissue envelope, distal femoral fractures occur predominately after low-energy trauma, e.g., falls and sprain injuries complicated by a high rate of comorbidity (60% female, older than 60 years). In young patients (60% male, younger than 40 years), high-energy trauma causes complex injury with comminuted and open fracture pattern. 30% of patients with distal femur fractures are polytraumatized.^2^ 40% had soft-tissue injuries. 10% had ligamentous lesions, 8% had meniscal lesions, 10% had dissected cartilage fragments and 15% had patella fractures. 38% of supracondylar/intercondylar distal femoral fractures have a coronal plane fracture.^2^–^5^ In recent literature, type C fractures are found in approximately 58% and open fractures in 27% of all cases.^1^ Debate continues around choice of implant for fixation of metaphyseal–diaphyseal fractures. In this retrospective study, we evaluated and compared clinical and radiological outcomes of distal femur fracture stablization using RN or LISS techniques.

## MATERIALS AND METHODS

Between January 2003 and December 2008, 115 patients with distal femoral fracture who had been treated by retrograde intramedullary (IM) Supracondylar Nailing System (n=59) Stryker Instruments, Kalamzoo, Michigan, USA (n=36) and T2 Femur nail, Stryker Instruments, Kalamzoo, Michigan, USA (n=23) or LISS plating (n=56) (Synthes, West Chester, Pennsylvania, USA) were evaluated in a retrospective study at a level 1 Trauma Center. Here 74 patients were men and 41 were women with a mean age of 54 years (range 17–89 years). The exclusion criteria to use a retrograde IM nail was a type C2 or C3 fracture. In these cases, a LISS plate osteosynthesis was performed.

The anteroposterior (AP) and lateral X-ray of the knee with distal femur were performed. CT scan was performed in all patients to assess displacement of fragments, intra-articular involvement, degree of comminution, as well as detection of coronal plane fractures that are difficult to identify on plane films.^6^ If impairment in perfusion or vascular injury is clinically diagnosed, diagnostic assessment using CT-angiography or conventional angiography is indicated and was performed in a total of 13 patients of the two cohort groups (*n*=115). In addition, X-ray view of the proximal femur and the hip joint (AP and lateral) was done to rule out a multilevel femur fracture in all cases as part of the diagnostic protocol.

### Operative procedure

LISS plate osteosynthesis: The patient was positioned supine on the radiolucent table with the knee flexed to avoid the typical hyperextension of the distal fragment caused by the pull of the gastrocnemius muscle.

After painting and draping a lateral skin incision at the distal femur aligned to Gerdy’s tubercle was made and extended deep through the soft tissues. The iliotibial band was exposed and incised in line with the skin incision. The vastus lateralis muscle was elevated from the intermuscular septum so far that the plate was positioned to the lateral condyle and the metaphyseal fracture was bridged in a no-touch technique. Care was taken not to disrupt the periosteum. In extra-articular fractures, a closed reduction was performed to avoid breaching of the periosteum. In intra-articular fractures, open reduction or miniopen reduction was performed to ensure anatomic reduction of the joint surface. In complex intra-articular fractures, a lateral parapatellar arthrotomy was performed to expose the articular surface as necessary after incision of the joint capsule. In these cases, the articular fracture was initially reduced and the condyle block was reconstructed and stabilized using temporary K-wire fixation. In complex C3 fractures, a tibial tubercle osteotomy of the tibia was performed to provide a sufficient exposure for the reduction of intra-articular fragments.

According to preoperative planning, a LISS plate long enough to comply with the principle of bridging osteosynthesis was selected, inserted under the vastus lateralis muscle, and slid proximally. Key success factor of the operation was the anatomic reduction of the articular fragments of the fracture and axis of the femoral shaft as well as correct implant position along the lateral femoral cortex. Plate position was considered correct if the distal monoaxially locking screws were parallel to the horizontal plane of the joint line on AP view. The plate position was secured by K-wires. Fracture reduction and implant positioning were verified by biplanar intraoperative fluoroscopic imaging. In extra-articular fractures four, and in intra-articular fractures five self-cutting, self-tapping, fixed-angle screws were inserted into the metaphyseal, distal fragment using the trocar system. In the femoral diaphysis, bicortical screws were preferentially inserted. All screws were tightened using a dynamometric screwdriver. K-wires were removed and the wound was irrigated. Confirmation of correct fracture reduction and hardware placement was achieved under intraoperative fluoroscopic control. Deep suction drains were placed, the wound was closed in layers, and sterile wound dressing was applied. The knee was moved through its range of motion, and ligamentous stability was tested under anesthesia.

Retrograde IM nailing: Preoperative planning and patient preparation was the same as described for LISS plating. For retrograde nailing (RN), either a T2 femur nail or an SCN retrograde IM nail was used (Stryker Instruments, Kalamzoo, Michigan, USA). In both nails, 5 mm interlocking screws were used. The SCN nail provides four distal interlocking screws and the possibility of compression and may be used for the stabilization of metaphyseal, distal femur fractures, whereas the T2 femur nail is regularly used for anterograde IM nailing and may also be used for retrograde IM nailing of distal femoral shaft fractures. The T2 femur nail provides three distal interlocking screws and the possibility of compression when used for retrograde IM nailing.

The patient was positioned supine on a radiolucent table. The fractured leg was positioned with knee flexion of 60 degrees to facilitate nail insertion. Following infrapatellar skin incision, direct transpatellar access to the knee joint was performed. In extra-articular fractures, percutaneous insertion of the retrograde nail was possible, whereas in comminuted intra-articular fractures additional lateral arthrotomy was required.

The insertion point was localized radiologically on the AP and lateral view in the intercondylar notch, anterior to Blumensaat’s line and in projection of the femoral shaft axis. Clinically, the correct insertion point was verified by positioning the K-wire anterior to the femoral insertion of the posterior cruciate ligament in the intercondylar notch. Following biplanar X-ray control, the K-wire was inserted into the medullary canal respecting a 7-degree valgus angle to the horizontal plane of the joint, and the cortex was opened using a 10-mm drill bit over the K-wire with a drill sleeve to protect from reaming debris. The K-wire was removed and replaced by a long guide wire. The latter was used to intubate the proximal fragment and positioned in the intramedullary canal proximal to the lesser trochanter. Limited reaming of the medullary canal was performed in 0.5 mm increments until cortical contact was appreciated. For final reaming, a reamer with a diameter of 2 mm larger than the selected nail diameter was used. The length of the nail was determined by measuring the guide wire, ensuring that the nail reaches proximally to the intertrochanteric region.

The retrograde nail was inserted under fluoroscopic control. Final position of the distal end of the nail was below the chondral surface in the subchondral bone of the distal femur. The distal interlocking screws were inserted using the aiming device and trocar. In the SCN nail, four 5-mm interlocking screws were used (proximal condyle screw 5 mm, two oblique locking screws 5 mm, distal condyle screw 5 mm). In the T2 femoral nail, three lateromedial interlocking screws were inserted. Whenever the dynamic compression option was utilized, the nail was inserted 1 cm deeper than the SCN. Insertion of an end cap locked the distal condyle interlocking screw and prevented screw loosening. Proximally, freehand insertion of two interlocking screws in AP direction was performed.

Careful postoperative treatment, with active and active-assisted physiotherapy where range of motion was limited by pain and discomfort, was initiated within 2 days following surgery. Mobilization was initiated with touch-down weight bearing under close supervision of a physical therapist. Twenty kilogram partial weight bearing was continued for approximately 6 weeks. Patients were mobilized out of bed with the use of two crutches, or a walking chair, and they learn to execute approximately 10 to 20 kg partial weight bearing by stepping on a weighing scale. The first postoperative X-ray control was performed following removal of wound drains. Clinical and radiological follow-up studies were performed after 6 and 12 weeks, and if osseous healing had not occurred also at 6, 9, and 12 months intervals. If delayed bone healing is observed or if patients are referred to our institution with manifestation of bony nonunion, revision surgery for treatment of delayed bone healing is performed. In atrophic delayed union or nonunion stability by the osteosynthesis and biological augmentation is performed using bone graft harvested from the iliac crest. In atrophic nonunion also bone morphogenic protein (BMP-7) is administered. In hypertrophic delayed union or nonunion bone graft is only added if cortical defect is observed. I hypertrophic nonunion, no BMP-7 is administered. Based on radiological fracture healing, gradual increase in weight bearing was permitted after 6 weeks, limited thereafter by pain and discomfort. Removal of hardware is recommended for LISS plate osteosynthesis if the implant is symptomatic, or earlier if local irritation of soft tissue occurs.

Osseous healing was defined radiographically as the presence of at least three of four healed cortices, with bridging callus formation and crossing trabeculae on AP and lateral radiographs. Clinical healing was defined as the absence of functional pain and local tenderness at the previous fracture site.

#### The knee and osteoarthritis outcome score was used to assess results

The KOOS score was calculated for each patient. A score of 100 to 90 points was considered to be an excellent result; 89 to 75 points a good result; 74 to 60 points a fair result; and <60 points a poor result. Criteria of the score include pain, symptoms, activity in daily living (ADL), sports and recreation function, and knee-related quality of life (QoL).^7^

### Statistical analysis

Data were analyzed with independent *t* tests as well as nonparametric Mann–Whitney, chi-square, and Fisher exact tests. The null hypothesis was that the two groups were similar. The experimental hypothesis was that the samples were from two different populations. All values represent means. A *P* value of <0.05 was considered to represent a significant finding.

## RESULTS

In the two cohort groups, mean age was 54 years (range 17-89 years). Mean follow-up was 14 months (range of 6–36 months) for the entire study group, with a mean follow-up of 15 months for the LISS group and 13 months for the RN group. Mechanism of injury was high-energy impact in 57% [(53% (n=31) RN, 67% (n=37) LISS) and low-energy injury in 43% (47% RN, 33% LISS)], respectively. Fractures were classified according to AO classification: there were 52 type A fractures (RN 31, LISS 21) and 63 type C fractures (RN 28, LISS 35). 32% (n=19) and 56% (n=31) were open fractures in the RN and LISS group, respectively.

**Figure 1:**
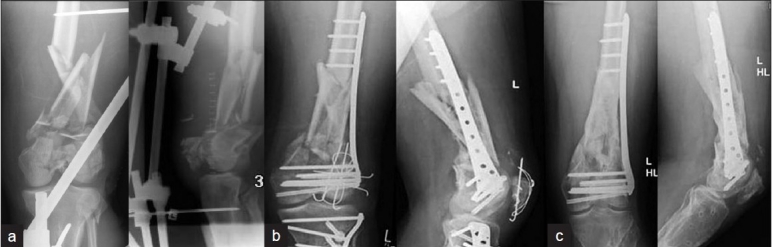
(a) Anteroposterior and lateral radiographs of left knee of a 38-year-old man with type C3 open distal femur fracture, patella fracture and proximal tibial fracture on the left leg; initial treatment with external fixator and temporary vacuum assisted closure. (b) Anteroposterior and lateral X-rays show definitive treatment with LISS plate after conditioning of soft tissues. (c) Nonunion developed and was treated with re-osteosynthesis, and application of osteogenic protein 1 (OP1, bone morphogenetic protein-7 (rhBMP-7)), and cancellous bone that resulted in osseous union. The Patellar and upper tibial implants were removed

Primary and definitive osteosynthesis was performed in 46% (n=53) of patients. In 54 % (n=62) the concept of damage control surgery^7^ was applied and the distal femoral fracture was stabilized using a temporary, joint spanning external fixator [Figure 1]. After a median of 7 days (range 3 to 12 days), conversion to the definitive osteosynthesis was performed and the external fixator removed. No specific selection criteria to use either the retrograde nail or the LISS plate were established for the conversion of temporary damage control stabilization into definitive fixation. The choice of implant for definitive osteosynthesis was dependent on both the fracture type and localization.

Clinical and radiographic evaluation demonstrated osseous healing within 6 months following RN and LISS plating in over 90% (n=104) of patients. Time to healing was not significantly different between the groups. In the RN group 5 out of 59 patients (9%) developed nonunion as no bony consolidation of the femoral fracture was observed 6 months after osteosynthesis. In the LISS group, nonunion was observed in 6 out of 56 patients (12%) [Table 1]. There was no statistically significant difference between the two groups for the development of nonunion. However, no statistically significant differences between the nail and the LISS group were found for the parameters time to osseous healing, rate of nonunion, and postoperative complications. Radiographic signs of healing correlated with clinical signs of healing, including the absence of pain or tenderness over the fracture site and the absence of pain with motion. The additional secondary bone grafting or bone substitute (BMP) was required, 3 months after the primary operation in four patients (7%) in RN group and six (12%) in LISS group.

**Table 1:** Bony healing and nonunion in the RN and LISS group

	Bony healing	Nonunion
RN (59)	54	5
LISS (56)	50	6

RN: Retrograde nailing, LISS: Less invasive stabilization on system

In patients who postoperatively complained about persisting joint pain with motion or weight bearing and whose conventional X-ray imaging demonstrated close proximity of the distal screws to the knee joint using conventional X-ray, a postoperative CT scan study was performed to determine intra-articular position of hardware. In the LISS group, we found intra-articular penetration of distal screws in four patients (7%) necessitating exchange of screws. In the RN group, no intra-articular hardware was found.

The following complications were treated: hematoma formation (one patient RN and three patients LISS), necessitating operative evacuation; superficial infection treated with surgical debridement and irrigation accompanied by high-dose intravenous antimicrobial therapy (one patient in RN and three patients in LISS group); deep infection treated with programmed wound lavage, vacuum therapy and intravenous high-dose antibiotics in two patient in LISS group). All four patients who developed hematoma had suffered closed fracture of the distal femur. All six patients who developed infection (four patient with superficial infection and two patients with deep infection had suffered open fracture of the distal femur. Indication for removal of retrograde i.m. nail was the symptomatic hardware. The retrograde i.m. nail had been removed in 36 patients till now.

**Figure 2:**
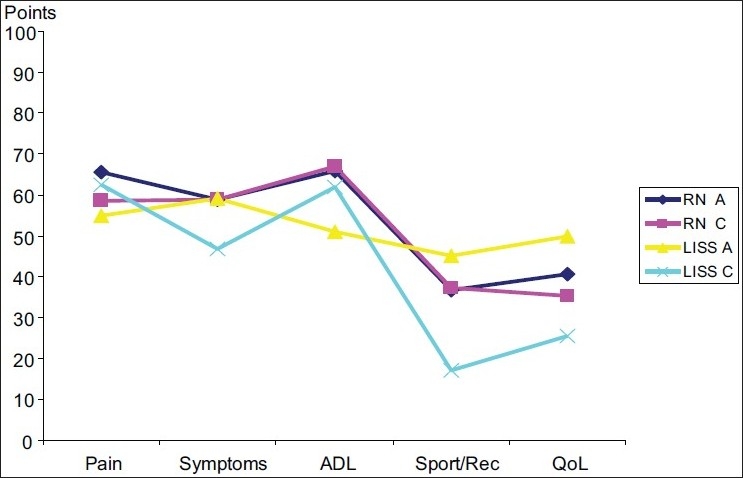
Graph showing functional outcomes using the KOOS score. In the LISS group, a significant reduction of KOOS score in type C fractures was observed. Criteria of the score include: pain, symptoms, activity in daily living (ADL), sports and recreation, and knee- related quality of life (QoL).

In type A fractures, KOOS^7^ scores of 263 (RN) and 260 (LISS), and in type C fractures 257 (RN) and 218 (LISS) were found. Statistical analysis of KOOS score results did not demonstrate significant differences between the groups for the accumulative result of KOOS and for the subgroups with pain symptoms, function in daily living, function in sports and recreation, or knee-related QoL [Figure 2].

## DISCUSSION

Distal femur fractures occur following high-energy impact in young patients often resulting in comminuted and open fractures, whereas low-energy injury is sufficient to cause distal femoral fractures in elderly patients with osteopenic or osteoporotic bone. For the treatment of distal femoral fractures, two major therapeutic principles can be employed: retrograde IM nailing or locking plate osteosynthesis. Both operative stabilizing systems follow the principle of biological osteosynthesis. Protection of soft-tissue envelope due to the minimally invasive approach and closed reduction techniques is better realized using IM. nailing. Aim of this study was to evaluate and compare clinical and radiological outcomes of distal femur fracture stabilization using RN or LISS plating.

In our retrospective study of 115 patients with distal femoral fracture, 59 patients were treated with RN and 56 patients with LISS plate osteosynthesis. No statistically significant differences between the nail and the LISS group were found so far for the parameters time to osseous healing, rate of nonunion and postoperative complications. The limitation of the study is retrospective nature of date analysis and the patients were not randomised to each group. Both stabilization systems, the RN and the locking plate osteosynthesis, require precise preoperative planning. Comprehension of fracture anatomy is essential for successful operative treatment of distal femoral fractures. In addition, advantage and limitations of each implant must be known and considered.

In the past, treatment of distal femur fractures was associated with high complication rates. Although implants and surgical techniques had improved, plate osteosynthesis and IM. nailing suffered from considerable rates of infection, nonunion, and malalignment. Attention to the soft-tissue envelope by introducing the concept of “biological” osteosynthesis and minimally invasive approaches resulted in decreased complication rates. Minimally invasive technique of osteosynthesis can be achieved by using two concepts: minimally invasive plating with an internal fixator – the LISS-DF (less invasive stabilization system – distal femur) – and even more by RN.

In this study, we focused on comparing treatment of distal femur fractures using RN or LISS plating and did not consider additional types of implants. Both surgical strategies employ indirect reduction techniques for the metaphyseal region, ensure anatomic reconstruction of the articular surface, and aim at restoration of axial alignment, rotation, and length of the femur.

### LISS - DF

The LISS system^9^ is an extramedullary, anatomically contoured internal fixator. Locking plate provides good fixation in osteoporotic bones in elderly patients.^10^–^12^ Previously, implants were selected depending on fracture type, whereas the LISS system can be universally applied for the treatment of all distal femoral fractures AO type A to C with the exception of AO type B Hoffa fractures, which are preferentially stabilized using lag screw osteosynthesis. The LISS plate provides enhanced distal screw fixation, even in osteoporotic bone, at the expense of more displacement at the fracture site. The rate of implant failure ranges between 5% and 10%.^2^,^12^,^15^ Compared with results published in the literature with nonunion rates following LISS plate osteosynthesis ranging from 1.6% to 6.1%,^12^–^14^ the high rate of nonunion in the LISS group found in our study may be attributed to the high incidence of open and comminuted C-type fractures in the cohort group. The incidence of infection following LISS plating of distal femoral fractures is reported with up to 4% of the cases.^2^,^12^,^15^

Main advantage of the anatomically precontoured LISS plate is soft-tissue protection using a limited approach and submuscular plate insertion, as well as percutaneous screw insertion facilitated by the aiming device. Fracture stabilization with the LISS system may render adequate reduction more difficult since the plate and the locking screws are not designed to approximate the fracture toward the plate.^9^ In fact, prior to plate fixation, fracture reduction has to be performed and completed. Once a locking screw has been placed through the plate into bone, this particular bone segment can no longer be manipulated by insertion of additional screws or by using compression devices. The sequence of screw placement has to be well planned to avoid fracture malreduction. Useful tool includes “no-hands” traction, femoral distractors, and percutaneous clamps.^9^ Distal screws are inserted perfectly parallel to the distal femoral joint line. Any angulation of screws in projection to the joint line may result in increased valgus or more detrimentally, in varus deviation.

The concept of bridging osteosynthesis implicates that the final fracture construct should be elastic and not too stiff to prevent formation of nonunion. Therefore, the screws should not be positioned too close to the fracture line in order to allow for elastic deformation of the plate–screw construct, thereby preventing the screws adjacent to the fracture from failing and being pulled out. The combination of a stiff plate, stiff screws, and fracture distraction is a formula for nonunion.

The size and contour of the plate may result in irritation of the iliotibial tract and may cause persistent pain. Symptomatic hardware has to be removed.^16^ Additional disadvantages include the complexity of insertion instruments, cross-threading of the screw–plate interface that is detrimental to biomechanical stability.

**Figure 3:**
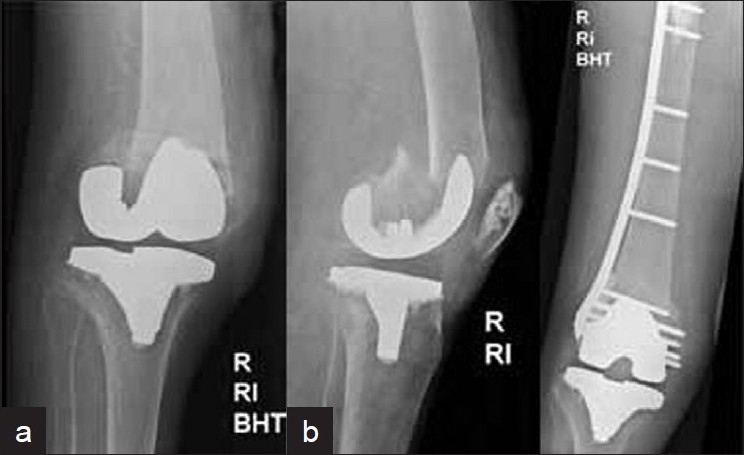
(a) Anteroposterior and lateral radiographs of right knee in a 88-year-old female with total hip and knee arthroplasty who fell and suffered a periprosthetic distal femur fracture. (b) Open reduction and internal fixation using LISS plate osteosynthesis was done and uneventful osseous healing as depicted was obtained

Indication for LISS plate osteosynthesis are as follows:^10^–^14^,^17^ Periprosthetic femur fracture around hip arthroplasty [Figure 3], open injury, short distal fragment, C2 and C3 fracture configuration, failed closed reduction with IM nailing, salvage implant for revision surgery and complicated situations. In our series, predominant indications for LISS plate osteosynthesis included grade III open injury, short distal fragment, and C2 and C3 fracture configuration.

### Retrograde nailing

Nailing provides favorable IM stability and can be successfully implanted in bilateral or multisegmental fractures of the lower extremity. In addition, a variety of distal femur fractures ranging from AO type A extra-articular metaphyseal, supracondylar, as well as intra-articular type C1 fractures can be stabilized. In these fractures, retrograde IM. nailing may be used and closed indirect fracture reduction is achieved by inserting the nail at a correct insertion point leaving the soft-tissue envelope intact.

**Figure 4:**
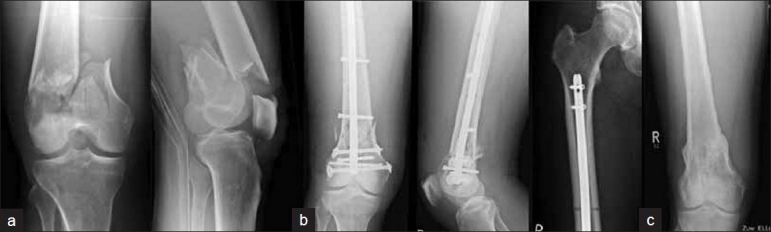
(a) Anteroposterior and lateral radiographs of right knee of a 65-year-old male who suffered a C1 distal femur fracture. (b) The fracture was stabilized using SCN retrograde nail. (c) Radiographs of knee following removal of nail depicting sound bony union and consolidation at fracture site

Intra-articular C1 fractures may also be treated with the retrograde nail but only if direct visualization and perfect reduction of the articular surface is possible [Figure 4]. Therefore, exposure of the joint line is required. In our series we excluded type C2 and C3 fractures for the use of IM nail osteosynthesis.

In contrast to the position of the distal screws in LISS plating which have to be positioned perfectly parallel to the joint line, distal interlocking screws of the retrograde nail have to be inserted at a valgus angle of approximately 7 degrees to the joint line. Only then the physiological valgus angle of the femoral condyle and the femoral shaft is respected and can be reconstructed.

Compared to plate fixation techniques, advantages of IM fixation systems, such as a retrograde femoral nail or an SCN, include soft-tissue protection due to small incision, decreased blood loss following limited exposure, “percutaneous” joint fixation, and the increased stability by IM fixation, load-sharing, and support of a long nail. Earlier biomechanic studies demonstrated that in distal supracondylar femur fractures, long nails reaching the intertrochanteric region provide increased fracture stability compared to short retrograde nails. The snug IM nail–bone fit improved the mechanical interaction between the femoral diaphysis and the nail.^18^

Indications for retrograde IM nailing for the treatment of distal femur fractures include:^18^–^20^

**Figure 5:**
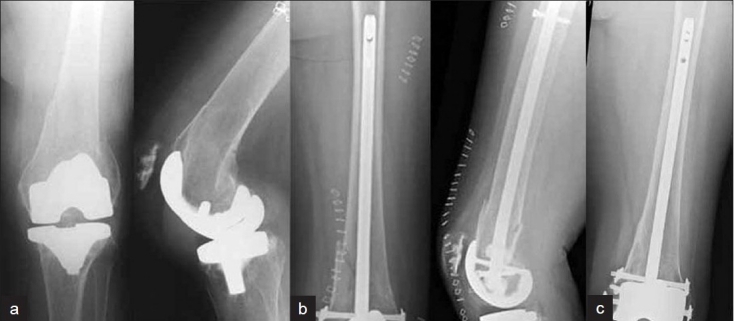
(a) Anteroposterior and lateral radiographs of a 60-year-old male with total knee arthroplasty who suffered a periprosthetic distal femur fracture. (b) Closed reduction and intramedullary stabilization was done using SCN (c) followup X-rays shows bony union

Distal femur fracture AO type A and C1, open wound around the knee, injury pattern, which requires supine position of patient with elevation of thorax and head, bilateral femur fractures, ipsilateral multilevel fracture, e.g., additional proximal femur fracture or combined femur and tibia fracture, periprosthetic fracture around a total knee arthroplasty [Figure 5], severe obesity.

In our series, predominate indications for osteosynthesis using the retrograde nail included distal femur fractures AO type A and C1, and open wound around the knee.

Contraindications for retrograde IM nailing include open epiphyseal cartilages, bone infection, pathologic fractures, total hip arthroplasty, and lung contusions. In infection, IM reaming and nail insertion may result in osseous distribution and spreading of bacteria and, therefore, IM nailing is not indicated in these cases.

Disadvantages of the nailing technique may be a lack of alignment control, retrocurvation, the intra-articular insertion, and perforation of joint cartilage and intra-articular distribution of reaming debris. Stability is limited if small diameter and short nails are inserted.

## CONCLUSION

Both retrograde IM nailing and LISS plating may be adequate treatment options for distal femur fractures. No differences in outcome between implants regarding fracture healing, nonunion, and infection were found. Locked plating may be utilized for all distal femur fractures including complex type C fractures, periprosthetic fractures, as well as osteoporotic fractures. IM nailing may provide favorable IM stability, may promote formation of circular and stable callus, and may be successfully implanted in bilateral or multisegmental fractures of the lower extremity as well as in extra-articular and type C1 fractures. However, both systems require precise preoperative planning and advanced surgical experience to reduce the risk of revision surgery. Clinical outcome may largely depend on surgical technique and rather than on the choice of implant. Multicenter studies with high numbers of patients are required to draw useful conclusions.
